# Plant chemical biology: are we meeting the promise?

**DOI:** 10.3389/fpls.2014.00455

**Published:** 2014-09-08

**Authors:** Glenn R. Hicks, Natasha V. Raikhel

**Affiliations:** Center for Plant Cell Biology, Department of Botany and Plant Sciences, University of CaliforniaRiverside, CA, USA

**Keywords:** chemical biology, chemical proteomics, small molecule, auxin, abscisic acid (ABA), jasmonate, endomembrane, vesicle

## Abstract

As an early adopter of plant chemical genetics to the study of endomembrane trafficking, we have observed the growth of small molecule approaches. Within the field, we often describe the strengths of the approach in a broad, generic manner, such as the ability to address redundancy and lethality. But, we are now in a much better position to evaluate the demonstrated value of the approach based on examples. In this perspective, we offer an assessment of chemical genetics in plants and where its applications may be of particular utility from the perspective of the cell biologist. Beyond this, we suggest areas to be addressed to provide broader access and enhance the effectiveness of small molecule approaches in plant biology.

## INTRODUCTION

The collaboration between plant biologists and chemists aimed at discovering new genes and protein functions has been accelerating over the past decade. In particular, the application of synthetic small molecules for basic discovery in plant systems has been growing and is becoming more sophisticated among basic researchers. There is now a modest number of plant-related research centers in the US, Belgium, Germany, and Sweden among other locations which house diverse and focused chemical collections, instruments, and expertise in screening for bioactive molecules and identification of their cognate targets. Such centers provide expertise and collaborative opportunities for plant biologists with varied interests who may not have access to chemical collections and other infrastructure. This is an important achievement given the broad range of plant biology, genomics, analytical and synthetic chemistry, proteomics, microscopy, and bioinformatics that may be required depending upon the specific project.

Our laboratory was an early adaptor of chemical genetics (i.e., the screening of small molecules and cognate target discovery) with an emphasis on the cell biology of plants. Along with our domestic and international colleagues, we have learned much about the benefits and challenges of the approach as a means to understand biological systems through the use of small bioactive molecules and other tools including “omics” approaches and biochemistry (i.e., chemical biology). As such we would like to offer several practical perspectives on the field from the viewpoint of cell biologists who have come to appreciate and value the power of multiple disciplines in solving problems. The concepts of plant chemical genetics have been reviewed previously by us and colleagues (Blackwell and Zhao, 2003; Kaschani and van der Hoorn, 2007; Walsh, 2007; De Rybel et al., 2009a,b; Hicks and Raikhel, 2009; Robert et al., 2009; Toth and van der Hoorn, 2009; Hicks and Raikhel, 2010, 2012; Ma and Robert, 2014). In addition, there are recent practical volumes covering many aspects of chemical biology in plant systems (Audenaert and Overvoorde, 2014; Hicks and Robert, 2014). The objective here is not to review the overall field; rather the focus will be on successful applications and the perspectives they offer at a practical level. We will then turn our focus to current challenges.

## WHAT IS THE VALUE OF CHEMICAL GENETICS TO PLANT BIOLOGY?

At a fundamental level, the key to chemical genetics as with conventional genetics is in generating recognizable phenotypes at the whole plant, organ, cell or subcellular level. Whereas genetics generates phenotypes based on mutations that, in turn, perturb protein expression or function, chemical genetics approaches generate phenotypes for the most-part by altering protein function directly. Given the variations possible within proteins in terms of amino acid number and sequence, post-translational modification, and secondary and tertiary structure, the potential target space for small molecules is vast. From the perspective of an academic plant biologist, however, the ability to access this chemical space is limited at a practical level by the availability of compounds and the resources to screen them. There are novel aspects to using a chemical approach. This includes access to an increased range of phenotypes compared to genetics alone since small molecules in principle are able to target multiple members of a protein family or essential proteins when applied at sub-lethal concentrations. From the cell biology perspective, the ability of reversible drug-like molecules to effectively relate cellular phenotypes to that at the organism level is very powerful as is the ability to study rapid cellular processes such as endomembrane trafficking or hormonal signaling (Hicks and Raikhel, 2012; Ma and Robert, 2014). One frequent claim is that chemical genetics approaches can permit the identification of genetically lethal single genes as well as functionally overlapping members of protein families. This is stated in the introductions of many manuscripts and reviews. Excluding industrial screens for pesticides, as a basic research community, we now approaching a decade of experience in plant chemical screening, target identification and the resulting biological knowledge. What do the results tell us?

## SOME SUCCESS STORIES

Although there have been chemical screens reported in the literature covering many areas of plant biology including endomembrane trafficking, hormone signaling, immunity, cell walls, and small RNAs (Hicks and Raikhel, 2012) with others appearing frequently [for example, see recently (Park et al., 2014; Paudyal et al., 2014; Xia et al., 2014)], we will focus in this review only on select molecules for which the cognate targets have been identified as these are the most informative for discussion here. Even within this limitation, the overall impact of chemical genetics in basic plant research has been widespread with some impressive successes (**Table 1**).

**Table 1:** Published small molecules with identified targets within gene families.

Small molecule	Target gene famliy	Function	Genes in family	Reference
Pyrabactin	PYR/PYRL	ABA preception	14	Park et al. (2009)
DAS 534	TIR/ABF	Auxin perception	5	Walsh et al. (2006)
Bikinin	GSK-3 kinase	Brassinosteroid signaling	10	De Rybel et al. (2009b)
Brassinazole	BIL4/BIL4-like	Possibly BR signaling	5	Yamagami et al. (2009)
Isoxaben	CESA	Cellulose biosynthesis	10	Desprez et al. (2002)
Gravicin	PGP/ABC transporter	Membrane transport	Up to 129	Rojas-Pierce et al. (2007)
BUM	PGP/ABC transporter	Membrane transport	Up to 129	Kim et al. (2010)
Jarin-1	GH3 amino acid conjugating enzymes	JA-Ile synthesis and signaling	19	Meesters et al. (2014)

There are multiple examples in hormone signaling. The transcriptional repression of auxin responses requires either TIR1 or one of its five homologs. By screening for resistance to a specific and highly potent picolinate-type auxin, DAS534, the homolog ABF5 was identified as a target (Walsh et al., 2006). ABF5 was not identified previously in mutant screens for 2,4D or IAA auxin resistance. The selectivity of DAS534 for ABF5 compared to other previously used auxins resulted in distinct plant phenotypes and permitted its identification as a target. Brassinosteroid perception and signaling occur via the plasma membrane BRI receptor system in which downstream signaling is activated by members of the GSK3-like kinase family of proteins. The compound bikinin was found to target a subset of seven of these kinases of which four were not implicated previously in brassinosteroid signaling (De Rybel et al., 2009b). In addition, the use of a brassinosteroid synthesis inhibitor, brassinazole, resulted in the identification of BIL4, a transmembrane protein among a family of five in *Arabidopsis* that may be involved in the control of cell elongation (Asami et al., 2003; Yamagami et al., 2009).

Perhaps the best example of groundbreaking work with small molecules was the use of a novel abscisic acid (ABA) agonist, pyrabactin, to identify the ABA receptor family (Park et al., 2009). Mutants resistant to pyrabactin were not resistant to ABA due to functional redundancy within the ABA receptor family. ABA insensitivity could only be achieved via multiple loss-of-function mutations within the so-call PYR/PYL receptors that belong to a large protein family known as the steroidogenic acute regulatory (StAR)-related lipid transfer (START) domain proteins. The receptors interact with PP2Cs releasing the negative regulation of Snf2-related kinase 2, activating ABA-responsive gene transcription. What quickly followed initial reports were crystallography studies detailing the molecular mechanisms of receptor binding and function (Melcher et al., 2009; Miyazono et al., 2009; Nishimura et al., 2009; Santiago et al., 2009; Yin et al., 2009). This was made possible by the ability to induce a detectable phenotype through the specific activity of pyrabactin for a PYR receptor among 14 within the family.

In the area of auxin biology, several molecules have been found to target auxin transporters. Gravicin is a chemical that inhibits gravitropism in *Arabidopsis* roots. The compound was subsequently found to target PGP19, a member of the super-family of ABC transporters with as many as129 members (Sanchez-Fernandez et al., 2001; Rea, 2007), which is involved in auxin transport. PGP19 also interacts with PIN auxin transporters (Rojas-Pierce et al., 2007). Another molecule (BUM) appears to target the PGP1 auxin transporter (Kim et al., 2010). Another example yet to be published is the identification of a specific exocyst component involved in recycling of PINs and other plasma membrane proteins. Very recently, there are exciting reports of specific protein family members involved in jasmonic acid-isoleucine conjugation and signaling (Meesters et al., 2014) as well as rationally designed jasmonate antagonists (Monte et al., 2014). Cognate targets for other molecules have been published for example in cell wall biosynthesis where certain members of the cellulose synthase family of proteins are targeted by isoxaben (Desprez et al., 2002; Somerville, 2006). While there may be additional examples of known small molecule targets, the examples cited here offer a perspective on plant chemical genetics. Namely, successful target identification is more likely to lead to results that are biologically meaningful which is essential goal of chemical biology. Anything short of this is ultimately a technical exercise.

## WHAT WORKS? WHAT DOES NOT?

What is clear from these examples is that success with small molecules generally lies in their ability to target one or more members of protein families. In such cases conventional loss-of-function mutants may not generate a detectable phenotype. Interestingly, in our examples the small molecules did not target all members of a conserved family. In the cases of auxin (Walsh et al., 2006) and ABA (Park et al., 2009) perception for which there are known ligands, the small molecules displayed altered target selectivity for receptors compared to known synthetic or native ligands. In other cases, inhibition of a subset of enzymes within a family (Asami et al., 2003; De Rybel et al., 2009b; Meesters et al., 2014) or inhibition of a class of transporters (Rojas-Pierce et al., 2007; Kim et al., 2010) resulted in distinct phenotypes. In some cases the phenotypes were scored in genetic screens for resistance to identify the cognate targets. So it seems that compounds should be promiscuous across a protein family in the interest of generating phenotypes, but not too much so. One hypothesis for this may be that small molecules acting too broadly may generate generalized growth phenotypes that confound genetic screens for targets. For example, perhaps such compounds are more likely to have off-target effects outside of a specific protein family making it difficult to identify the cognate target. But overall, the trend indicates that the power of chemical biology as practiced lies in the ability to generate phenotypes among protein families. This is highly significant since in *Arabidopsis* one third or more of the genes are in families (Hicks and Raikhel, 2012).

What about essential single copy genes? In principle, small molecules should be able to target essential proteins/genes in a dose-dependent manner, in other words by treatment at sub-lethal concentrations. So why is this not included in our examples from plants? The answer may be in the approaches used to identify targets. The most reliable approach used to identify cognate targets in plants is to screen for altered sensitivity to compounds. This takes advantage of EMS-induced mutations (or T-DNA loss-of-function insertions) and the well-developed genetics and genomics available in *Arabidopsis*. The ability to sequence whole genomes from pooled mapping populations has greatly increased the speed of EMS mutation identification (Schneeberger et al., 2009; Austin et al., 2011; Hartwig et al., 2012). Among our examples, with the exception of cases where the targets were deduced based on activity within known pathways (De Rybel et al., 2009b; Meesters et al., 2014) or molecules were rationally designed to target a receptor complex (Monte et al., 2014), forward EMS screens were used to identify targets. The dilemma of using forward EMS mutant screens for resistance is that recessive loss-of-function mutations in essential genes will not be represented or recoverable from a screening population. Thus, unless one is fortunate to identify a relatively rare gain-of-function (dominant) mutant displaying small molecule resistance, the probability of identifying an essential gene as a cognate target is relatively small. Thus, the recoverable targets favor members of protein families from which one or few members targeted by a small molecule result in a phenotype that is not lethal and can be scored for mapping. It cannot be determined from the literature how many reported compounds were pursued for targets genetically, or, of those attempted, how many mutant screens did not result in targets. But this may explain, at least in part, why in addition to the effort required a relative minority of novel compounds shown to be bioactive in plants have reported targets. Overall then, the power of small molecules when combined with EMS mutants appear to be in identifying the functions of protein families and subsets of their members.

## THE CASE FOR CHEMICAL PROTEOMICS

For cases outside of protein families these observations would argue for alternative approaches for the identification of targets that are less biased against essential genes. These alternative methods are based on the affinity of small molecules for their protein targets [discussed in (Hicks and Raikhel, 2010)]. This set of approaches is also known as chemical proteomics (Futamura et al., 2013). In principle, coupling bioactive molecules to a solid matrix or bead permits direct affinity purification of potential targets. Such methods have been in use for drug target discovery for some time (Rix and Superti-Furga, 2009), yet have not gained much traction among plant biologists. This may be due to several factors including ready collaborations with synthetic chemists capable of producing tagged molecules. Contributors to the uncertainty of these approaches include (1) the feasibility of producing appropriate tagged bioactive molecules based on structure-activity relationships (SAR), (2) the often unknown affinity of proteins for their small molecule ligand and their abundance, (3) the unknown intracellular or organellar location of the target, (4) whether the cognate target will bind to the ligand in protein extracts *in vitro* due to ionic strength and pH for example, and (5) whether a protein complex is required for binding. An additional important reason that alternative approaches may have not been explored fully is that most plant biologists are trained as geneticists who are comfortable especially with *Arabidopsis* resistance screens and mutation mapping. A shift to other approaches requires additional training and collaboration. These issues require a careful initial characterization of compound potency and the analysis of structural analogs, including those with moieties such as amines that are suitable for coupling to biotin or other tags without greatly diminishing activity. This requires early collaboration with synthetic chemists before focusing exclusively on a small molecule.

On the optimistic side, mass spectroscopy instruments have greatly increased in sensitivity making feasible the association between compounds and protein targets even at extremely low levels. This in itself addresses many of the issues cited above. More recently, we have utilized an approach known as drug affinity responsive target stability (DARTS; Lomenick et al., 2009, 2011a,b). The approach is based on the principle that small molecule binding to a target site can stabilize a protein thereby decreasing its sensitivity to protease digestion. The small molecule-protein interaction can then be detected using mass spectroscopy (i.e., chemical proteomics). The advantage of the approach is that is not dependent upon producing a modified ligand. Rather the active compound can be used. Based upon several factors cited above, this approach will not work in all cases or even most cases, but it is simple to establish the assay and is therefore a simple first approach to target identification. Another important aspect of chemical proteomics approaches is the ability to detect the interactions of many proteins with a small molecule (Rix and Superti-Furga, 2009; Futamura et al., 2013). This can be used to detect the range of desired targets as well as off-target effects and should be applicable to plant systems. This has proven useful especially among pharmaceuticals where a profile of targets can address drug specificity and off-target effects. Even within large gene families chemical proteomics has been used to profile drug selectivity (for recent example, see Ku et al., 2014). A group of compounds could be examined rather quickly for interactions, with the most promising warranting further investigation. If there is an antibody to a suspected target then the approach can be made more focused, increasing the probability of success. Other possible methods not requiring tagged molecules include methods designed to co-elute proteins with active small molecules (Chan et al., 2012). Although still being developed and adopted for plants, these approaches to assay for direct protein-ligand interaction are an important option prior to or in combination with genetics for target identification and should provide broader access to essential genes.

## FINAL PERSPECTIVES

Briefly, there are several other noteworthy areas that have gained from chemical biology from the cell biology perspective. One is the realization that with screens that are rapid and simple, it has been possible to find small molecules that directly perturb vesicle trafficking (Drakakaki et al., 2011) which impacts other processes such a cell wall biosynthesis (Park et al., 2014). As it turns out, virtually all such compounds result in scorable growth or developmental phenotypes to identify cognate targets. Furthermore, rapid mapping by sequencing requires the scoring of far fewer recombinants than conventional mapping, so it is now much more feasible to map mutations by scoring at the microscopy level. A perhaps overlooked, but powerful benefit of chemical biology is the ability to score for intracellular phenotypes directly then link the phenotypes directly to their corresponding developmental consequences. Given the available large selection of fluorescent protein-tagged intracellular markers, it is efficient to score small molecules across a selection of intracellular markers. It is then possible to focus on desired intracellular and plant developmental phenotypes. The contrasting option is to use genetics. This would require generating and screening mutagenized populations across the same selected marker lines followed by screening each population for intracellular defects, a daunting task even assuming that the same range of phenotypes could be obtained by the two approaches. Another area that we have noted previously is the need for continued enhancement in plants of automated screening utilizing image and video analysis (Hicks and Raikhel, 2009). This includes the tracking of plant development at macro and micro levels [for several recent examples, see (Tisne et al., 2013; Sozzani et al., 2014)], vesicle movement related to basic mechanisms, plant immunity and development within tissues such as the meristem (Salomon et al., 2010; Tataw et al., 2013; Ung et al., 2013; Beck et al., 2014). In quantifying aspects of vesicle or organelle diameter, velocity, and directionality we can learn about the regulation of dynamic subcellular compartments in real time. In this manner small molecules can be used to directly modulate intracellular trafficking in a quantifiable manner to dissect pathway function and regulation.

Our final perspective on chemical genetics is that as the field is maturing, it is becoming critical for labs to focus on the biology and what can be learned using this approach. That is, the focus should be increasingly on the biology aspects of chemical biology. It is not enough to simply find new compounds with interesting bioactivities. Rather, we have to push harder to demonstrate that biological insight has been gained in each study. This is a characteristic of each successful example published in high profile journals and what we should strive for as a community.

## Conflict of Interest Statement

The authors declare that the research was conducted in the absence of any commercial or financial relationships that could be construed as a potential conflict of interest.

## References

[B1] Asami T. Nakano T. Nakashita H. Sekimata K. Shimada Y. Yoshida S. (2003). The influence of chemical genetics on plant science: shedding light on functions and mechanism of action of brassinosteroids using biosynthesis inhibitors. *J. Plant Growth Regul.* 22 336–349 10.1007/s00344-003-0065-0 14676973

[B2] Audenaert D. Overvoorde P. (2014). *Plant Chemical Biology.* Hoboken, NJ: John Wilsey & Sons

[B3] Austin R. S. Vidaurre D. Stamatiou G. Breit R. Provart N. J. Bonetta D. (2011). Next-generation mapping of *Arabidopsis* genes. *Plant J.* 67 715–725 10.1111/j.1365-313X.2011.04619.x 21518053

[B4] Beck M. Zhou J. Faulkner C. Robatzek S. (2014). High-throughput imaging of plant immune responses. *Methods Mol. Biol.* 1127 67–80 10.1007/978-1-62703-986-4_5 24643552

[B5] Blackwell H. E. Zhao Y. (2003). Chemical genetic approaches to plant biology. *Plant Physiol.* 133 448–455 10.1104/pp.103.031138 14555772 PMC1540336

[B6] Chan J. N. Vuckovic D. Sleno L. Olsen J. B. Pogoutse O. Havugimana P. (2012). Target identification by chromatographic co-elution: monitoring of drug-protein interactions without immobilization or chemical derivatization. *Mol. Cell. Proteomics* 11 M111.016642. 10.1074/mcp.M111.016642 PMC3394955 22357554

[B7] De Rybel B. Audenaert D. Beeckman T. Kepinski S. (2009a). The past, present, and future of chemical biology in auxin research. *ACS Chem. Biol.* 4 987–998 10.1021/cb9001624 19736989

[B8] De Rybel B. Audenaert D. Vert G. Rozhon W. Mayerhofer J. Peelman F. (2009b). Chemical inhibition of a subset of *Arabidopsis* thaliana GSK3-like kinases activates brassinosteroid signaling. *Chem. Biol.* 16 594–604 10.1016/j.chembiol.2009.04.008 19549598 PMC4854203

[B9] Desprez T. Vernhettes S. Fagard M. Refregier G. Desnos T. Aletti E. (2002). Resistance against herbicide isoxaben and cellulose deficiency caused by distinct mutations in same cellulose synthase isoform CESA6. *Plant Physiol.* 128 482–490 10.1104/pp.010822 11842152 PMC148911

[B10] Drakakaki G. Robert S. Szatmari A. M. Brown M. Q. Nagawa S. Van Damme D. (2011). Clusters of bioactive compounds target dynamic endomembrane networks in vivo. *Proc. Natl. Acad. Sci. U.S.A.* 108 17850–17855 10.1073/pnas.1108581108 22006339 PMC3203817

[B11] Futamura Y. Muroi M. Osada H. (2013). Target identification of small molecules based on chemical biology approaches. *Mol. Biosyst.* 9 897–914 10.1039/c2mb25468a 23354001

[B12] Hartwig B. James G. V. Konrad K. Schneeberger K. Turck F. (2012). Fast isogenic mapping-by-sequencing of ethyl methanesulfonate-induced mutant bulks. *Plant Physiol.* 160 591–600 10.1104/pp.112.200311 22837357 PMC3461541

[B13] Hicks G. R. Raikhel N. V. (2009). Opportunities and challenges in plant chemical biology. *Nat. Chem. Biol.* 5 268–272 10.1038/nchembio0509-268 19377447

[B14] Hicks G. R. Raikhel N. V. (2010). Advances in dissecting endomembrane trafficking with small molecules. *Curr. Opin. Plant Biol.* 13 706–713 10.1016/j.pbi.2010.08.008 20851666

[B15] Hicks G. R. Raikhel N. V. (2012). Small molecules present large opportunities in plant biology. *Annu. Rev. Plant Biol.* 63 261–282 10.1146/annurev-arplant-042811-105456 22404475

[B16] Hicks G. R. Robert S. (2014). *Plant Chemical Genomics.* New York: Springer 10.1007/978-1-62703-592-7

[B17] Kaschani F. van der Hoorn R. (2007). Small molecule approaches in plants. *Curr. Opin. Chem. Biol.* 11 88–98 10.1016/j.cbpa.2006.11.038 17208036

[B18] Kim J. Y. Henrichs S. Bailly A. Vincenzetti V. Sovero V. Mancuso S. (2010). Identification of an ABCB/P-glycoprotein-specific inhibitor of auxin transport by chemical genomics. *J. Biol. Chem.* 285 23309–23317 10.1074/jbc.M110.105981 20472555 PMC2906323

[B19] Ku X. Heinzlmeir S. Helm D. Medard G. Kuster B. (2014). New affinity probe targeting VEGF receptors for kinase inhibitor selectivity profiling by chemical proteomics. *J. Proteome Res.* 13 2445–2452 10.1021/pr401247t 24712744

[B20] Lomenick B. Hao R. Jonai N. Chin R. M. Aghajan M. Warburton S. (2009). Target identification using drug affinity responsive target stability (DARTS). *Proc. Natl. Acad. Sci. U.S.A.* 106 21984–21989 10.1073/pnas.0910040106 19995983 PMC2789755

[B21] Lomenick B. Jung G. Wohlschlegel J. A. Huang J. (2011a). Target identification using drug affinity responsive target stability (DARTS). *Curr. Protoc. Chem. Biol.* 3 163–180 10.1002/9780470559277.ch110180 22229126 PMC3251962

[B22] Lomenick B. Olsen R. W. Huang J. (2011b). Identification of direct protein targets of small molecules. *ACS Chem. Biol.* 6 34–46 10.1021/cb100294v 21077692 PMC3031183

[B23] Ma Q. Robert S. (2014). Auxin biology revealed by small molecules. *Physiol. Plant* 151 25–42 10.1111/ppl.12128 24252105

[B24] Meesters C. Mönig T. Oeljeklaus J. Krahn D. Westfall C. S. Hause B. (2014). A chemical inhibitor of jasmonate signaling targets JAR1 in *Arabidopsis thaliana*. *Nat. Chem. Biol.* 10.1038/nchembio.1591 [Epub ahead of print] 25129030

[B25] Melcher K. Ng L. M. Zhou X. E. Soon F. F. Xu Y. Suino-Powell K. M. (2009). A gate-latch-lock mechanism for hormone signalling by abscisic acid receptors. *Nature* 462 602–608 10.1038/nature08613 19898420 PMC2810868

[B26] Miyazono K. Miyakawa T. Sawano Y. Kubota K. Kang H. J. Asano A. (2009). Structural basis of abscisic acid signalling. *Nature* 462 609–614 10.1038/nature08583 19855379

[B27] Monte I. Hamberg M. Chini A. Gimenez S. García G. Porzel A. (2014). Rational design of a ligand-based antagonist of jasmonate perception. *Nat. Chem. Biol.* 10 671–676 10.1038/nchembio.1575 24997606

[B28] Nishimura N. Hitomi K. Arvai A. S. Rambo R. P. Hitomi C. Cutler S. R. (2009). Structural mechanism of abscisic acid binding and signaling by dimeric PYR1. *Science* 326 1373–1379 10.1126/science.1181829 19933100 PMC2835493

[B29] Park E. Diaz-Moreno S. M. Davis D. J. Wilkop T. E. Bulone V. Drakakaki G. (2014). Endosidin 7 specifically arrests late cytokinesis and inhibits callose biosynthesis revealing distinct trafficking events during cell plate maturation. *Plant Physiol*. 165 1019–1034 10.1104/pp.114.241497 24858949 PMC4081319

[B30] Park S. Y. Fung P. Nishimura N. Jensen D. R. Fujii H. Zhao Y. (2009). Abscisic acid inhibits type 2C protein phosphatases via the PYR/PYL family of START proteins. *Science* 324 1068–1071 10.1126/science.1173041 19407142 PMC2827199

[B31] Paudyal R. Jamaluddin A. Warren J. P. Doyle S. M. Robert S. Warriner S. L. (2014). Trafficking modulator TENin1 inhibits endocytosis, causes endomembrane protein accumulation at the pre-vacuolar compartment and impairs gravitropic response in *Arabidopsis thaliana*. *Biochem. J*. 460 177–185 10.1042/BJ20131136 24654932 PMC4100570

[B32] Rea P. A. (2007). Plant ATP-binding cassette transporters. *Annu. Rev. Plant Biol.* 58 347–375 10.1146/annurev.arplant.57.032905.105406 17263663

[B33] Rix U. Superti-Furga G. (2009). Target profiling of small molecules by chemical proteomics. *Nat. Chem. Biol.* 5 616–624 10.1038/nchembio.216 19690537

[B34] Robert S. Raikhel N. V. Hicks G. R. (2009). Powerful partners: *Arabidopsis* and chemical genomics. *Arabidopsis Book* 7 e0109. 10.1199/tab.0109 PMC3243329 22303245

[B35] Rojas-Pierce M. Titapiwatanakun B. Sohn E.-J. Fang F. Larive C. Blakeslee J. (2007). *Arabidopsis* P-Glycoprotein19 participates in the inhibition of gravitropism by Gravacin. *Chem. Biol.* 14 1366–1376 10.1016/j.chembiol.2007.10.014 18096505

[B36] Salomon S. Grunewald D. Stuber K. Schaaf S. Maclean D. Schulze-Lefert P. (2010). High-throughput confocal imaging of intact live tissue enables quantification of membrane trafficking in *Arabidopsis*. *Plant Physiol.* 154 1096–1104 10.1104/pp.110.160325 20841454 PMC2971591

[B37] Sanchez-Fernandez R. Davies T. G. Coleman J. O. Rea P. A. (2001). The *Arabidopsis* thaliana ABC protein superfamily, a complete inventory. *J. Biol. Chem.* 276 30231–30244 10.1074/jbc.M103104200 11346655

[B38] Santiago J. Dupeux F. Round A. Antoni R. Park S. Y. Jamin M. (2009). The abscisic acid receptor PYR1 in complex with abscisic acid. *Nature* 462 665–668 10.1038/nature08591 19898494

[B39] Schneeberger K. Ossowski S. Lanz C. Juul T. Petersen A. H. Nielsen K. L. (2009). SHOREmap: simultaneous mapping and mutation identification by deep sequencing. *Nat. Methods* 6 550–551 10.1038/nmeth0809-550 19644454

[B40] Somerville C. (2006). Cellulose synthesis in higher plants. *Annu. Rev. Cell Dev. Biol.* 22 53–78 10.1146/annurev.cellbio.22.022206.160206 16824006

[B41] Sozzani R. Busch W. Spalding E. P. Benfey P. N. (2014). Advanced imaging techniques for the study of plant growth and development. *Trends Plant Sci.* 19 304–310 10.1016/j.tplants.2013.12.003 24434036 PMC4008707

[B42] Tataw O. M. Reddy G. V. Keogh E. J. Roy-Chowdhury A. K. (2013). Quantitative analysis of live-cell growth at the shoot apex of *Arabidopsis thaliana*: algorithms for feature measurement and temporal alignment. *IEEE/ACM Trans. Comput. Biol. Bioinform*. 10 1150–1161 10.1109/TCBB.2013.64 24384704

[B43] Tisne S. Serrand Y. Bach L. Gilbault E. Ben Ameur R. Balasse H. (2013). Phenoscope: an automated large-scale phenotyping platform offering high spatial homogeneity. *Plant J.* 74 534–544 10.1111/tpj.12131 23452317

[B44] Toth R. van der Hoorn R. A. (2009). Emerging principles in plant chemical genetics. *Trends Plant Sci.* 15 81–88 10.1016/j.tplants.2009.11.005 20036182

[B45] Ung N. Brown M. Q. Hicks G. R. Raikhel N. V. (2013). An approach to quantify endomembrane dynamics in pollen utilizing bioactive chemicals. *Mol. Plant* 6 1202–1213 10.1093/mp/sss092 23118478 PMC7105205

[B46] Walsh T. A. (2007). The emerging field of chemical genetics: potential applications for pesticide discovery. *Pest Manag. Sci.* 63 1165–1171 10.1002/ps.1452 17912687

[B47] Walsh T. A. Neal R. Merlo A. O. Honma M. Hicks G. R. Wolff K. (2006). Mutations in an auxin receptor homolog AFB5 and in SGT1b confer resistance to synthetic picolinate auxins and not to 2,4-dichlorophenoxyacetic acid or indole-3-acetic acid in *Arabidopsis*. *Plant Physiol.* 142 542–552 10.1104/pp.106.085969 16920877 PMC1586033

[B48] Xia Y. Lei L. Brabham C. Stork J. Strickland J. Ladak A. (2014). Acetobixan, an inhibitor of cellulose synthesis identified by microbial bioprospecting. *PLoS ONE* 9:e95245 10.1371/journal.pone.0095245 PMC3991599 24748166

[B49] Yamagami A. Nakazawa M. Matsui M. Tujimoto M. Sakuta M. Asami T. (2009). Chemical genetics reveal the novel transmembrane protein BIL4, which mediates plant cell elongation in brassinosteroid signaling. *Biosci. Biotechnol. Biochem.* 73 415–421 10.1271/bbb.80752 19202280

[B50] Yin P. Fan H. Hao Q. Yuan X. Wu D. Pang Y. (2009). Structural insights into the mechanism of abscisic acid signaling by PYL proteins. *Nat. Struct. Mol. Biol.* 16 1230–1236 10.1038/nsmb.1730 19893533

